# Paediatric DNA methylation profile scores: a systematic review and open-source atlas

**DOI:** 10.1016/j.ebiom.2026.106300

**Published:** 2026-05-22

**Authors:** Isabel K. Schuurmans, Serena Defina, A.P.C. Hermans, Alexander Neumann, Matthew Suderman, Paul Yousefi, Janine F. Felix, Charlotte A.M. Cecil

**Affiliations:** a Department of Child and Adolescent Psychiatry and Psychology, Erasmus MC University Medical Center Rotterdam, Rotterdam, the Netherlands; b The Generation R Study Group, Erasmus MC, University Medical Center Rotterdam, Rotterdam, the Netherlands; c MRC Integrative Epidemiology Unit, Population Health Sciences, Bristol Medical School, University of Bristol, Bristol, UK; d NIHR Bristol Biomedical Research Centre, University Hospitals Bristol and Weston NHS Foundation Trust and University of Bristol, UK; e Department of Pediatrics, Erasmus MC, University Medical Center Rotterdam, Rotterdam, the Netherlands; f Department of Epidemiology, Erasmus MC University Medical Center Rotterdam, Rotterdam, the Netherlands; g Molecular Epidemiology, Department of Biomedical Data Sciences, Leiden University Medical Center, Leiden, the Netherlands

**Keywords:** Epigenetics, Polygenic risk score, Prediction, Paediatric, Biomarker

## Abstract

Methylation profile scores (MPSs) have emerged as a useful tool to index exposures, biological states, or disease-related patterns, by aggregating information across many DNA methylation (DNAm) sites into a single score per individual. Although most MPSs to date have been trained and validated in adults, their use in early life (<18 years) is rapidly gaining momentum, as more cohorts profile DNAm during development, offering a window on exposures and vulnerability before disease onset. Here we systematically review early-life MPS studies (n = 119), covering 828 MPSs that span a wide range of exposures and traits. Overall, studies show substantial heterogeneity in design and reporting. Also, studies frequently apply MPSs trained in data from mismatched age groups, tissues, array or platforms, and ancestries, which may influence MPS performance. To address these limitations, we introduce DEMETRA (indepthlab-demetra.share.connect.posit.cloud), a searchable atlas of early-life MPSs to help users identify and select scores best suited to their study.

## Introduction

Epigenetic processes regulate gene activity dynamically in response to genetic and environmental factors, beginning in utero,^1^^,^^2^ without changing the underlying gene sequence. These processes play a key role in healthy development and ageing^3^ and are implicated in the emergence of a range of mental and physical conditions, including neurodevelopmental and psychiatric disorders, neurological and cardiometabolic disease, and cancer.^4^, ^5^, ^6^, ^7^, ^8^, ^9^, ^10^, ^11^, ^12^ Among different epigenetic mechanisms, DNA methylation (DNAm) is one of the best characterised in humans. DNAm involves the addition of methyl groups to specific DNA base pairs (mostly cytosine–guanine dinucleotides; CpGs) to tag, stabilise, or regulate genomic regions.^13^ DNAm can now be measured at high throughput and reasonable cost, enabling profiling in large population cohorts. Because of these characteristics, DNAm has emerged as a valuable biological system for studying gene-environment interplay in health and disease.

An extensive body of research has profiled associations between DNAm and genetic variants, environmental exposures, traits, and health outcomes,^14^ most often through epigenome-wide association studies (EWAS) that estimate associations across hundreds of thousands of CpGs using array-based platforms, or across millions of sites when whole-genome approaches are used. Analogous to GWAS, these studies show that DNAm signals are often polyepigenetic: while individual CpGs typically only exert small effects in isolation, combining information across CpGs explains more variation in exposures or outcomes. Because of this, the field is increasingly turning to methylation profile scores (MPSs), which aggregate the effects of multiple CpG sites into a single score per individual, akin to polygenic scores (PGSs) in genetics. MPSs (also known as epigenetic risk scores, episcores, or episignatures)^15^, ^16^, ^17^, ^18^, ^19^, ^20^, ^21^ can be trained to capture more than an individual’s genetic susceptibility, including environmental exposures (e.g., smoking, diet), biological states (e.g., low-grade inflammation indexed by C-reactive protein),^22^ and complex traits and diseases (e.g., tumours, asthma). They can therefore serve several purposes, from proxying missing or inaccurately measured variables, to improving comparability across different studies by using the same score, to enhancing risk prediction models and quantifying biological responses to interventions (e.g., epigenetic clocks in anti-ageing research).^23^^,^^24^ MPSs are also beginning to be applied to clinical and translational settings, such as improving diagnostic yield for Mendelian disorders^25^ and in forensic profiling.^26^

**Fig. 1:**
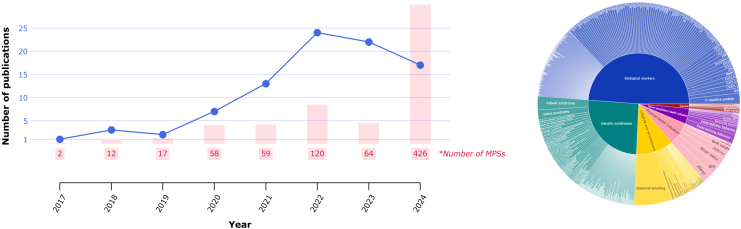
**Overview methylation profile score phenotypes.** On the left, we see the number of publications and MPSs published over the years. On the right, we see what phenotypes are most often inferred by MPSs in early life, coloured by category. **Left**: 2025 not included as not complete yet. Graph includes number of publications (blue) and number of MPSs (red). **Right**: Categories of MPS include biological markers (blue), genetic syndromes (green), lifestyle and environment (yellow), physical health indicators (pink), neuro-psychiatric health indicators (purple), and cancer (red). An interactive graph can be found at: indepthlab-demetra.share.connect.posit.cloud.

However, MPSs so far have mainly been confined to adult research. In other words, they are typically *trained* (i.e., with CpG weights derived in adult discovery datasets) and *applied* (i.e., calculated and evaluated in independent adult samples) within adult populations.^15^, ^16^, ^17^, ^18^, ^19^, ^20^, ^21^ Interest in bringing these MPSs into the context of early life is rising (Fig. 1) in view of its importance for lifelong mental and physical health.^27^ In particular, MPSs in early life provide researchers a means to (i) capture genetic and environmental influences at a time when they interact most profoundly on biology, with relevance for understanding long-term embedding of risk, and (ii) assess DNAm *before* the onset of most diseases and treatments, helping to minimise reverse causation while enhancing opportunities for prevention.

Yet, because development represents a fundamentally different biological context, applying MPSs in early life is not equivalent to applying them in adulthood. DNAm patterns change across infancy, childhood, and adolescence^28^; exposure patterns interact with immune, cardiometabolic, and neurodevelopmental systems that are still maturing; and several periods reflect sensitive windows in which exposures may express stronger effects. Beyond biology, early-life MPS studies face additional methodological challenges. Exposures and phenotypes differ in meaning across developmental stages^29^; tissue choice is often more constrained and dependent on age (e.g., cord blood at birth vs saliva or buccal cells in childhood)^30^; and working with paediatric populations often implies more extensive ethical consideration. Together, these factors mean that adult-trained MPSs cannot be assumed to generalise or reflect the same underlying constructs in developmental settings. Although several commentaries and reviews have already summarised adult MPS research,^15^, ^16^, ^17^, ^18^, ^19^, ^20^, ^21^ comparable efforts to identify, contextualise, and synthesise early-life MPS research are lacking.

Here, we provide the first systematic review of MPSs trained or applied in early life. Specifically, (i) we identify early-life MPSs by providing a bird’s-eye view on which MPSs have been used to date in early life studies, (ii) we contextualise early-life MPSs by summarising what factors may influence their *performance* (i.e., how well an MPS captures or predicts the target phenotype) and *generalisability* (i.e., how well an MPS performs when applied outside the context in which it was trained), and (iii) we introduce the Developmental Methylation Risk Atlas (DEMETRA) as a searchable catalogue of MPSs trained or applied in early life to help users select the most optimal MPSs for their research question and target sample. We conclude by outlining key considerations for extending MPSs across the life course and priorities for future work.

## Search strategy and selection criteria

We conducted a systematic review, last updated on 25 July 2025, to identify studies training or applying MPSs in early life (≤18 yrs), conducted across three major databases: EMBASE, Google Scholar, Web of Science, and MEDLINE. Detailed search terms and methods are provided in the Supplementary Methods. We queried for “methylation profile score” (and various synonyms) and “paediatric” (and various synonyms). We excluded studies focusing solely on epigenetic age, epigenetic clocks, or telomere length, as these have already been reviewed extensively elsewhere.^31^^,^^32^ Our search retrieved 406 unique articles (Supplementary Table S1), which underwent a two-step screening process. Two reviewers (IKS, APCH) independently screened abstracts and then full texts, with disagreements resolved by discussion or, if needed, by consultation with a senior reviewer (CAMC). This process yielded 119 studies that met the eligibility criteria.

Across these 119 studies, we extracted information on all reported MPSs and their applications. Fifty-six percent of publications reported only one MPS, whereas 13% reported more than ten (maximum 278 MPSs in a single study). In total, we identified 828 unique MPSs, inferring 357 distinct phenotypes (Fig. 1). Forty-three percent of these phenotypes were linked to more than one MPS, either because they were independently trained in different studies or because multiple methodological strategies were explored within the same publication. A comprehensive summary of the characteristics of the 119 included studies and 828 identified MPSs is provided in Table 1.

**Table 1:** Summary of results.

	n (%)/M [range]		n (%)		n (%)
Included studies	119	Developmental period		Platform	
Design		Birth	179 (22%)	Multiple	413 (50%)
Population-based	27 (23%)	Very early childhood	7 (1%)	450 K	68 (8%)
Case-control	62 (52%)	Early childhood	5 (1%)	EPICv1	322 (39%)
Cohort study	24 (20%)	Mid childhood	322 (39%)	EPICv2	2 (0.2%)
Convenience sample	2 (2%)	Late childhood	44 (5%)	WGBS	14 (2%)
Twin study	3 (3%)	Adolescence	47 (6%)	Nanopore sequencing	8 (1%)
Randomised controlled trial	1 (1%)	Childhood and adolescence	149 (18%)	Ancestry	
Number of MPSs calculated	1 [1; 278]	Not reported	63 (8%)	African	2 (0.2%)
		Tissue		European/White	540 (65%)
Methylation profile scores, N	828	Blood-derived	648 (78%)	Latino/Hispanic	6 (0.7%)
Category		Saliva and buccal	145 (17%)	Multiple/Admixed	58 (7%)
Genetic syndromes	211 (25%)	Other (e.g., placenta, tumour)	33 (4%)	Not reported	222 (27%)
Cancer	12 (1%)	Multiple	1 (0.1%)		
Biological markers	404 (49%)	Not reported	1 (0.1%)		
Lifestyle and environment	99 (12%)				
Physical health indicators	61 (7%)				
Neuro-psychiatric health indicators	41 (5%)				

M = median.

## Which MPSs are most commonly used in early life?

The most frequently inferred phenotype was maternal smoking (58 MPSs), followed by Kabuki syndrome (20 MPSs), asthma, allergy, and c-reactive protein (each 14 MPSs), and finally Sotos syndrome, BMI, internalizing/externalizing behaviour, and atopic status (10–13 MPSs each). Generally, the MPSs can be organised into four broader categories, as described below. A full catalogue of the MPSs included in this review is accessible through the **Developmental Methylation Risk Atlas (DEMETRA)** (see Section 5) or in Supplementary Table S1.

### Biological and physiological markers

The largest category of MPSs across early-life studies (404 MPSs; 49% of all scores, across 12 publications) involves those inferring biological and physiological states, such as circulating proteins and inflammatory markers that are not themselves clinical outcomes. Many of these MPSs were originally trained in adults,^33^^,^^34^ but have since been applied to younger cohorts to proxy biological variables when they are not directly measured, such as inferring c-reactive protein levels from DNAm when serum c-reactive protein is unavailable. Protein-based MPSs and inflammation MPSs illustrate how DNAm can be used as a scalable, blood-based proxy for complex molecular phenotypes, with potential applications in identifying individuals at elevated risk for poor health (i.e., risk stratification) and testing whether DNAm mediates the effects of early exposures on later health outcomes (i.e., mechanistic pathways).

### Rare diseases and syndromes

The second-largest category (211 MPSs; 25% of all scores, across 58 publications) focuses on inferring rare conditions for diagnostic applications. Most of these involve genetic syndromes, with MPSs classifying 116 distinct conditions, including Kabuki and Sotos syndromes. Here, MPSs are used to increase the diagnostic yield for conditions that are driven by pathogenic variants but that are also characterised by heterogeneous and overlapping clinical presentations, sometimes with ambiguous genetic findings. These diagnostic MPSs are applied cross-sectionally to classify individuals as having or not having a given condition based on their current DNAm profile, rather than predicting future disease incidence. Major resources in this domain include the EpiSign™ platform^25^ and EpigenCentral.^35^ A smaller subset of diagnostic MPSs are used to infer cancer, particularly paediatric brain tumours: 12 MPSs (1% of scores) across 11 publications were used to aid tumour subtype identification, for instance using the Heidelberg classifier for DNAm-based reclassification.^36^ If further validated, these diagnostic MPSs may in future complement genetic testing and support more precise diagnoses and tailored clinical care, although their clinical utility remains to be established in clinical settings.

### Environmental and lifestyle exposures

A third major category comprises MPSs of environmental and lifestyle exposures (99 MPSs; 12% of scores, across 25 publications), inferring 30 unique phenotypes including smoking, poverty, and pollution. Maternal prenatal smoking MPSs are by far the most common. They are typically calculated in offspring cord blood or peripheral blood collected in childhood, and are widely used both as proxies when (maternal) self-reported smoking exposure is not available or as biological readouts of cumulative exposure in offspring.^37^, ^38^, ^39^, ^40^ These exposure-focused MPSs are particularly relevant for early life research, where retrospective exposure assessment is often imperfect and biomarkers can provide more objective or personalised measures, even among children with similar reported exposure levels. For example, two infants exposed to comparable amounts of maternal smoking may show different DNAm patterns depending on interactions with co-occurring factors such as maternal stress, nutrition, or genetic susceptibility.

### Physical and neuropsychiatric health phenotypes

A smaller set of MPSs (61 MPSs; 7%) was used to infer clinical disease states or diagnoses. Specifically, we identified eight physical health phenotypes in paediatric samples, including allergy, asthma, and BMI, while 41 MPSs (5%) targeted 11 neuropsychiatric phenotypes, such as bipolar disorder and schizophrenia. These scores are typically used to probe whether DNAm patterns associated with adult disease liability or severity are detectable earlier in development, or to predict childhood- or adolescent-onset conditions from perinatal or early-life DNAm. For example, MPSs trained using newborn and childhood DNAm have been used to predict the development of childhood asthma.^41^

Across these categories, the broad aims of MPSs are to proxy unmeasured variables, to assess predictive utility beyond direct measurements, to act as potential mediators in exposure–outcome relationships or to aid diagnosis. However, the ability of MPSs to fulfil these roles in early life may be affected by several challenges in the developmental setting that can affect both their predictive accuracy and interpretability.

## What factors may influence MPSs performance in early life?

Independent of the specific variable inferred, the successful use of MPSs in early life depends on how well the data used for MPS development matches the context of application. Four factors may be especially relevant for determining generalisability: developmental timing, tissue, technical platform, and ancestry. These factors shape both how MPSs behave and how their results should be interpreted and applied broadly across different MPSs.

### Developmental timing

In our review, MPSs were applied to samples ranging from birth through adolescence (most studies focusing on mid-childhood; ≈ 6–9 years; 39%). Early life is a period characterised by non-linear and more rapid biological change as compared to adulthood.^28^ These programmed, non-linear changes can alter the CpG-phenotype relations that underlie MPS algorithms and reduce their temporal reliability. For example, MPSs that are trained in adults show only moderate test–retest reliability in children (r ≈ 0.42–0.51), while test-retest reliability is much higher in adults (r > 0.80).^42^ This could imply that the same MPSs may be more time-specific when used in childhood as compared to adulthood.

Moreover, MPSs trained at one timepoint do not necessarily generalise to other stages of development. The vast majority (87%) of MPSs used in early life were trained in a different age group, most commonly from adult models (52%). Unsurprisingly, when transferred to early life, most adult-based scores show limited generalisability. For instance, of 14 adult-trained protein-based MPSs, 10 were significantly associated with protein levels in middle-aged adults and 9 in young adults aged 24 years, whereas none were significant at p < .05 in 9-year-olds.^34^ Similar attenuation was observed for an MPS of C-reactive protein, with correlations between the MPS and the phenotype declining from around r = 0.5 in midlife to r = 0.2 or lower in childhood.^33^ These examples highlight the need to evaluate age-generalisability before applying existing MPSs to developmental contexts.

Longitudinal DNAm data provide an opportunity to directly assess how MPSs behave over time and whether their predictive value varies across developmental stages. This may also allow us to identify *sensitive windows*, in which certain MPSs are more strongly linked to later outcomes.^43^ Generally, DNAm is thought to be more plastic and environmentally responsive in early life than in adulthood,^42^ meaning some DNAm differences may be transient, with early deviations later normalising. Without repeated measures, such transient shifts risk being misinterpreted as permanent,^31^ complicating interpretation and clinical use.

### Tissue and cell composition

Among the studies reviewed, MPSs were most often applied using DNAm measured in peripheral or whole blood (63%). Other tissues sources include saliva (15%), cord blood (7%), dried blood spots (3%), blood clots (3%), buccal cells (2%), placenta (2%), tumour cells (1%), nasal epithelial cells (1%), leukocytes (<1%), cervical cells (<1%), urine (<1%), and combinations of tissues (e.g., whole blood and hippocampal progenitor cells; <1%). Because DNAm is largely cell-type specific,^44^, ^45^, ^46^ comparability across tissues is often limited, meaning that an MPS trained in one tissue does not automatically generalise to another.

Despite this, 56% of reviewed MPSs were applied in a different tissue than the one they were originally trained in. The most common mismatch involved applying blood-derived MPSs to saliva, a scenario in which cross-tissue evaluations have shown limited reliability (intraclass correlation coefficients: 0.69 for cognitive ability, 0.58 for c-reactive protein, 0.54 for BMI).^30^ Similar patterns have been observed when cord- or blood-derived MPSs are applied to buccal cells.^47^

Several factors contribute to these mismatches in paediatric research. First, the tissues sources available for DNAm profiling change with age: cord blood is typically collected at birth, whereas saliva or buccal cells are more common in later childhood, particularly in high-risk samples, due to their non-invasive collection. Even within the same tissue type, developmental shifts in cell type composition can impact MPS reproducibility over time. For example, in blood, the relative proportions of lymphoid and myeloid cells change from birth through adolescence,^48^^,^^49^ and cell types such as nucleated red blood cells and haematopoietic stem cells are present at birth but virtually absent later.^50^^,^^51^

Efforts to address these issues include developing new DNAm reference panels that better capture cell-type dynamics across the lifespan^52^ as well as constructing MPSs that are specifically designed to be robust across tissues, for example, by selecting CpGs with high cross-tissue reproducibility^53^; but the extent to which these methods fully mitigate tissue challenges remains unclear.

### Technical platform and preprocessing pipelines

Technical aspects are another key factor that may influence MPS generalisability. While whole-genome bisulfite sequencing (WGBS) is the gold standard for DNAm measurement, assessing all 28 million available CpGs,^54^ its cost and computational demands limit its use in large-scale studies (only one WGBS-based study was included in this review). Most MPS studies reviewed here rely instead on arrays that only assess a subset of CpGs, such as the Illumina’s BeadChip microarrays, including the 450 K array (∼450,000 CpGs; 8% of included MPSs),^55^ the EPICv1 array (∼850,000 CpGs; 39%),^56^ and the more recent EPICv2 (∼900,000 CpGs; 2 MPSs).^57^ Newer arrays typically include more CpGs that are detected with higher quality, which can improve MPS performance (e.g., for CHD2 syndrome^58^).

This rapid evolution of arrays introduces challenges when MPSs trained in one platform are applied to data from another platform, a situation that occurred in 84% of reviewed MPSs. This can be problematic, as MPS performance is typically reduced when the training and application datasets used different arrays.^59^ Strategies to address this include restricting analyses to overlapping CpGs across arrays, using principal-component-based MPSs that rely on shared variance across CpGs,^60^ or imputing missing CpGs using methods such as random forest (missForest),^61^ PCA-based imputation (imputePCA),^47^ or k-nearest neighbours.^62^ These approaches can reduce, but likely do not fully remove, discrepancies arising from array differences.

Beyond array type, several other technical factors may also influence the generalisability of MPSs. Different normalisation pipelines (e.g., quantile normalisation, functional normalisation, noob correction)^63^ are known to change DNAm estimates and, in turn, may affect MPS performance.^19^ DNAm can also be analysed as either beta values (0–1 proportions) or M-values (logit-transformed), with some evidence that M-values offer improved predictive accuracy in certain contexts.^64^ Additional sources of variation include differences in sample collection, processing, and laboratory conditions. Transparent reporting of these technical details is therefore essential for both MPS developers and users, in order to facilitate interpretation, comparison, and reproducibility of results.

### Ancestry and diversity

Ancestry is associated with DNAm patterns through both genetic background and socially patterned determinants of health, including income, neighbourhood environment, and diet.^65^ These factors contribute to population differences in DNAm patterns, which in turn influence MPS generalisability. Similar to other areas of genomics, current MPS research is skewed towards White/European ancestry populations: 65% of MPSs in our review were trained and applied in such samples, 8% were based on other or mixed ancestries, and ancestry information was missing for 27%. This lack of diversity and incomplete reporting mirrors challenges in the epigenetic clock literature^66^ and raises concerns about equity, as MPSs trained primarily in White/European samples may perform poorly in minoritized populations, thereby risking to exacerbate existing health disparities.

Among the relatively few non-European ancestry studies, training and application datasets often differed in ancestry. These often involved MPSs trained in European ancestry cohorts but applied to non-European populations, echoing what is also seen in polygenic score research.^67^ An illustrative example comes from a maternal smoking MPS trained in cord blood DNAm in a European cohort, which showed a strong association with self-reported prenatal smoking in European offspring but not in a South Asian cohort.^39^ However, the same MPS was associated with lower birth size in both populations, with even larger effects in South Asian offspring. This suggests that the MPS may capture biologically relevant downstream processes even when the primary exposure phenotype is not equivalently captured across groups and points to a complex interplay between ancestry, reporting biases, and downstream phenotypes.

These ancestry-related mismatches can be mitigated through several strategies, including training MPSs while excluding CpGs strongly associated with ancestry^68^ and training ancestry-specific or multi-ancestry MPSs. It should be noted, however, that these approaches trade off MPS performance within subpopulations against generalisability and risk discarding CpGs genuinely associated with exposures or outcomes that differ between populations. Ultimately, more diverse and representative training datasets will be essential to ensure that MPSs lessen, rather than exacerbate, health inequalities.

## DEMETRA: a Developmental Methylation Risk Atlas to facilitate MPS selection for early life studies

To support the field and make our systematic review maximally reusable, we developed **DEMETRA (Developmental Methylation Risk Atlas)**, an online resource that catalogues MPSs trained or applied in early life (indepthlab-demetra.share.connect.posit.cloud). DEMETRA compiles a number of key characteristics and meta-data for each MPS, including:

• The inferred phenotype (e.g., exposure, biological marker, genetic syndromes, health outcome)
• The life stage(s) and tissues in which it was trained and applied
• Key methodological choices (training dataset, platform, basic model type)
• Links to the original publications.

Users can query DEMETRA amongst others by inferred category, inferred phenotype, and developmental period, enabling rapid identification of existing MPSs relevant to a given research question and highlighting areas where no suitable MPSs have yet been applied to early life. DEMETRA is designed to complement existing resources such as the Polygenic Score Catalogue,^69^ EpiSign,^25^ and EpigenCentral,^35^ but with a dedicated focus on early life and developmental applications of MPSs. A comparison with existing resources is provided in Supplementary Table S2.

Moreover, DEMETRA includes an upload function that allows researchers to register their own MPSs directly in the database, promoting standardised reporting and community-driven curation. When applying MPS in research, we recommend following the minimal reporting guidelines outlined in Supplementary Box 1 of the Supplementary Information.

## Discussion and outstanding questions

Our systematic review reveals a rapidly growing, but methodologically heterogeneous landscape of MPSs applications in early life. We identified 828 unique MPSs across 119 studies, spanning rare conditions, environmental exposures as well as biological and health outcome categories. While this body of work demonstrates the flexibility and promise of MPSs in early life, it also highlights important gaps. Most early life applications still rely on MPSs that are derived from adults, in a different tissue, and in Eurocentric samples. Further, key aspects of study design and preprocessing are often underreported, limiting comparability and synthesis across studies.

For the broader genomics community, MPSs in early life provide a powerful testbed for questions of generalisability and context-specificity. Developmental timing, tissue, technical platform, and ancestry all appear as potential axes along which MPS performance can vary. Early life is particularly informative in this respect, because of rapid biological change, evolving tissue availability, and shorter exposure histories which could naturally stress-test the robustness of predictive models. Insights gained from early life research may thus inform best practices for MPS trained and applied across the life course.

Moreover, beyond the factors discussed, methodological choices in MPS construction may also influence performance and interpretability. First, MPSs typically capture a mixture of genetically and environmentally driven variation in DNAm, and whether this is desirable depends on the research question. For example, retaining genetically influenced CpGs may improve disease prediction, whereas minimising genetic confounding may be preferable when targeting environmental signals. In this context, methylation quantitative trait loci (mQTLs) can inform CpG selection,^70^ for instance by prioritising or excluding genetically influenced sites, although such strategies have not yet been systematically applied in paediatric research. Alternatively, genetic influences can be accounted for by adjusting for polygenic scores of the same phenotype, as done by Odintsova et al.^47^ Second, MPSs can be developed using different methodological frameworks, including approaches based on EWAS summary statistics or machine learning models (see Yousefi et al.),^21^ and the method used may affect how the MPS is behaving or can be interpreted. Finally, principal component-based MPSs may improve robustness to platform and batch effects by capturing shared variation across CpGs, but at the cost of reduced interpretability of the included individual CpGs.^60^ Future research should identify best methodological practices specifically for MPSs as applied in early life.

Looking ahead, several priorities stand out. First, expanding and combining early-life DNAm datasets will be crucial to enable robust construction and validation of developmentally-tuned MPSs. Longitudinal designs enable stratification across biologically meaningful developmental periods, while advanced statistical approaches, such as Gaussian Process Regression, may further improve modelling of non-linear relationships between age and methylation. Importantly, this should be done across diverse ancestries. Currently, 65% of early-life MPSs in our review were trained and applied in White/European samples. Without deliberate efforts to diversify training and validation cohorts, there is a risk that MPSs will perform best in already well-represented groups, thereby reinforcing health disparities. Second, greater methodological standardisation and transparent reporting are needed. This includes clear documentation of training and application contexts, normalisation and preprocessing pipelines, and validation strategies, as we also recommend in Supplementary Box 1 in the Supplement. Currently, substantial heterogeneity in reported performance metrics (e.g., R^2^, AUC, variance explained, sensitivity/specificity) precludes formal meta-analysis. DEMETRA marks a first step toward more consistent reporting, by standardising key metadata including phenotype, tissue, platform, and performance metrics, which authors are prompted to provide when uploading a new MPS. However, a broader community uptake of reporting guidelines will be essential to enable systematic evaluation of what works and under what conditions. Third, if these priorities are addressed, improvements in MPS performance may follow. Although clinical or population-level implementation is not yet within reach, it will be interesting to evaluate whether, with sufficient gains in predictive accuracy, MPSs could support early-life risk stratification.

While this mini-review focuses on early life, the ultimate goal is to integrate MPSs into life-course frameworks, in which epigenetic predictors are trained, validated, and applied across multiple ages and tissues. Such frameworks will be necessary to capture how vulnerability is shaped over time and to leverage the potential of MPSs as a new tool to support early risk detection, disease prevention, and health promotion from the start of life.

**Research in context**

**Evidence before this study**

Methylation profile scores (MPSs), also referred to as epigenetic risk scores, episcores, or episignatures, have been widely developed and applied in adult populations to index environmental exposures, biological states, and disease-related processes using DNA methylation data. Prior narrative reviews and commentaries have summarised methodological approaches and applications of MPSs in adults, including their use for exposure proxying, risk prediction, and diagnostic support. However, before this study, no systematic review had comprehensively identified, catalogued, and evaluated the use of MPSs in early life (≤18 years). Although individual early-life studies existed, the overall evidence base had not been systematically synthesised, and no pooled estimates or structured assessment of generalisability across developmental contexts had been undertaken.

**Added value of this study**

This study provides the first systematic review of MPS applications in early life, identifying 828 unique MPSs across 119 studies and spanning 357 inferred phenotypes. By synthesising this literature, we demonstrate substantial heterogeneity in study design, reporting, and analytical choices, and show that most early-life applications rely on MPSs trained in adults, different tissues, array or platforms, or ancestries. We identify four key dimensions (developmental timing, tissue, technical platform, and ancestry) that could influence MPS performance and generalisability in early life. In addition, we introduce DEMETRA, a publicly accessible, searchable Developmental Methylation Risk Atlas that systematically catalogues early-life MPSs along with their training and application contexts. DEMETRA provides a practical tool to support informed MPS selection and highlights gaps where developmentally appropriate scores are lacking.

**Implications of all the available evidence**

Taken together, existing evidence and our findings indicate that early life represents a distinct biological and methodological context in which MPSs cannot be assumed to perform equivalently to adulthood. Applying mismatched MPSs in paediatric samples risks reduced validity and misinterpretation. Our results underscore the need for developmentally tuned MPS training, improved reporting standards, and greater ancestral diversity in epigenetic research. For human health research, early-life MPSs offer unique opportunities to capture biological embedding of exposures before disease onset and to study mechanisms linking early environments to later health. DEMETRA provides an infrastructure to support more rigorous and transparent use of MPSs in paediatric and life-course research, facilitating progress toward prevention-oriented and equitable applications of epigenetic risk profiling.

## Contributors

IKS, SD and CAMC conceptualised the study. IKS and APCH curated the data. IKS contributed to methodology and project administration. SD performed formal analyses, visualisation and prepared software and resources. IKS and CAMC wrote the original draft. SD, APCH, AN, MS, PY, and JFF reviewed and edited. CAMC supervised the project. All authors read and approved the final version of the manuscript.

## Declaration of interests

Paul Yousfi received personal fees (honoraria) from the University of Galway and the University of Edinburgh for lectures, presentations, or educational activities. The authors declare no other competing interests.

## Appendix A Supplementary data

Supplementary Box 1 and Tables S2 and S3

Supplementary Table S1

Graphical abstract
