# Supplementary material for: Paediatric DNA methylation profile scores: a systematic review and open-source atlas
Source: eBioMedicine. 2026 May 22;128:106300. doi: 10.1016/j.ebiom.2026.106300 (PMC13224112; doi:10.1016/j.ebiom.2026.106300)
Supplement: Supplementary Box 1 and Tables S2 and S3 [file mmc1.docx]

**Paediatric DNA methylation profile scores: a systematic review and open-source atlas**

*Isabel K. Schuurmans*^1,2^*, Serena Defina*^1,2^*, A. P. C. Hermans*^1,2^*, Alexander Neumann*^1,2^*, Matthew Suderman^3,4^, Paul Yousefi^3,4^, Janine F. Felix*^2,5^*, Charlotte A.M. Cecil*^1,2,6,7^

^1^ Department of Child and Adolescent Psychiatry and Psychology, Erasmus MC University Medical Center Rotterdam, Rotterdam, the Netherlands  ^2^ The Generation R Study Group, Erasmus MC, University Medical Center Rotterdam, Rotterdam, the Netherlands

^3^ MRC Integrative Epidemiology Unit, Population Health Sciences, Bristol Medical School, University of Bristol, Bristol, UK

^4^ NIHR Bristol Biomedical Research Centre, University Hospitals Bristol and Weston NHS

Foundation Trust and University of Bristol

^5^ Department of Pediatrics, Erasmus MC, University Medical Center Rotterdam, Rotterdam, the Netherlands

^6^ Department of Epidemiology, Erasmus MC University Medical Center Rotterdam, Rotterdam, the Netherlands

^7^ Molecular Epidemiology, Department of Biomedical Data Sciences, Leiden University Medical Center, Leiden, the Netherlands

**Short title:** early life methylation profile scores

**Correspondence:** Isabel K. Schuurmans, Department of Child and Adolescent Psychiatry and Psychology, Erasmus MC University Medical Center Rotterdam, PO Box 2040, 3000 CA Rotterdam, the Netherlands. Email: i.schuurmans@erasmusmc.nl

**Box 1. Recommendation for reporting MPSs in research**

| **1. Publication information**  **1.1 Title.** Provide the full title of the publication where the MPS was introduced or used.  **1.2 Authors.** List all authors involved in the development or application of the MPS. Include primary contact information.  **1.3 DOI.** Include the DOI for the publication.  **1.4 Journal.** Specify the journal in which the MPS was published.  **1.5 Publication date.** State date of publication.  **2. MPS information**  **2.1 Phenotype.** Clearly define the phenotype or health outcome the MPS is designed to predict (e.g., asthma, allergy, neuropsychiatric health etc.)  **2.2 Sample size.** Report the total sample size used for constructing the MPS, including specific details on cases vs. controls if applicable.  **2.3 Based on.** Indicate the foundation for the MPS (e.g., pre-established MPS, newly developed via published summary statistics [semi-supervised], or newly developed with raw individual-level data).  **2.4 Array type.** Specify the DNA methylation array used for data collection (e.g., 450K, EPICv1, EPICv2).  **2.5 Tissue source.** List the biological tissue from which the samples were obtained (e.g., blood, saliva, cord blood, buccal swabs).  **2.6 Age range.** Provide the age range of the subjects, including minimum and maximum age. If possible, include mean, median and standard deviation for better understanding of the population.  **2.7 Ancestry.** Describe the ancestry of the study population (e.g., European, Asian, African).  **3. Technical information** (*only* when newly developed based on published summary statistics or based on raw individual-level data)  **3.1 Methodology.** Specify the method used for MPS development (e.g., linear regression, lasso). Include any preprocessing steps applied to the methylation data.  **3.2 Predictive performance.** Report the predictive performance metrics of the MPS, such as AUC (Area Under the Curve), explained variance, or other relevant measures.  **3.3 Link to code/MPS weights.** Provide a link to the publicly available code used to compute the MPS (if open access is permitted), and include the MPS weights if possible to allow for reproducibility.  **4. Source information** (*only* for pre-established MPS or when based on published summary statistics)  **4.1 Reference to source dataset.** Provide citations and references to the original dataset or datasets used for developing the MPS, including relevant metadata.  **4.2 Reference DOI.** Include the DOI for the dataset or algorithm used for reproducibility.  **4.3 Matching criteria.** Clearly state whether the application of the MPS matches the original dataset on key parameters such as array type, tissue type, age range, and ancestry. If not, describe any potential limitations of the MPS due to these differences. |
| --- |

**Supplementary Table 2**

| Feature | DEMETRA | PGS Catalog | NGDC Ewas Atlas | meffonym | EpiScore / EpiSign |
| --- | --- | --- | --- | --- | --- |
| Data type | Methylation profile scores | Polygenic scores | CpG associations | Methylation profile scores | Methylation profile scores |
| Primary role | Atlas to identify the most appropriate MPS for your dataset | Atlas to identify the most appropriate PGS for your dataset | Tool to characterize other exposures/traits your CpG of interest is associated to | Computing the MPS in your own dataset | Computing the MPS in your own dataset |
| Unit | Meta-data available MPSs | Meta-data available PGSs | CpG-level statistics between phenotype and DNAm | Model objects to calculate MPSs in a dataset | Model objects to calculate MPSs in a dataset |
| Early-life focus | Yes | Not specifically | Not specifically | Not specifically | Not specifically |
| Meta-data | Publication ID, number of CpGs, how trained, sample type, sample size, age period, tissue, array, ancestry | Publication ID, number of variants, ancestry | Publication ID | Publication ID, tissue | None |
| Searchable interface | Yes | Yes | Yes | No | No |
| Score calculation | No | Yes | No | Yes | Partial |

**Systematic review**

We performed a systematic review, with a primary search completed on September 9, 2024, and updated on July 25, 2025. The search was conducted across three major databases: EMBASE, Google Scholar, and MEDLINE (Supplementary Table 2). We queried for “methylation profile score” (and various synonyms) and “pediatric” (and various synonyms); the full search terms used to identify relevant studies can be found below. Duplicate entries were removed, leaving 406 articles, which underwent a two-step screening process, with first abstract review and next full text review. Two reviewers independently assessed abstracts, excluding 287 articles. To be eligible for inclusion, studies needed to meet the following criteria: (i) include DNAm data, (ii) calculate a methylation profile score (defined as a score derived from at least two CpG sites), and (iii) target children as the study population (i.e., the MPS must have been applied to participants under 18 years of age). Studies were excluded if they met any of the following criteria: (i) preprints, reviews or conference posters, (ii) case studies, (iii) studies utilizing methylation profile scores based on global methylation across the entire genome or a gene-based average, or (iv) studies focusing on epigenetic age, epigenetic clocks, or telomere length, as these have already been reviewed extensively elsewhere. Ultimately, 119 studies met the criteria and were included in this review.

*Supplementary Table 3***.** Overview of eligible articles

| **Database searched** | **Platform** | **Years of coverage** | **Records** | **Records after duplicates removed** |
| --- | --- | --- | --- | --- |
| Medline ALL | Ovid | 1946 - Present | 174 | 171 |
| Embase | Embase.com | 1971 - Present | 338 | 179 |
| Web of Science Core Collection* | Web of Knowledge | 1975 - Present | 154 | 28 |
| Additional Search Engines: Google Scholar** (50 top-ranked) | | | 50 | 28 |
| **Total** | | | **716** | **406** |

*Science Citation Index Expanded (1975-present) ; Social Sciences Citation Index (1975-present) ; Arts & Humanities Citation Index (1975-present) ; Conference Proceedings Citation Index- Science (1990-present) ; Conference Proceedings Citation Index- Social Science & Humanities (1990-present) ; Emerging Sources Citation Index (2005-present)

**Google Scholar was searched via "Publish or Perish" to download the results in EndNote.

No other database limits were used than those specified in the search strategies

**Search terms**

***Embase***

(('DNA methylation'/de AND 'scoring system'/de) OR (episcore* OR episign* OR ((methylation* OR epigenetic) NEAR/6 (risk) NEAR/6 (score* OR measure)) OR ((methylation*) NEAR/6 (profile) NEAR/6 (score*)) OR ((methylation* OR epigenetic*) NEAR/3 (proxy)) OR ((DNAm OR DNA-methylation* OR polyepigenetic OR poly-epigenetic OR aggregate-methylation*) NEAR/3 (score*)) OR aggregate-DNAm OR methscor* OR methylation-score* OR (MPS AND methylat*)):ab,ti,kw) **AND** (juvenile/exp OR (juvenil* OR adolescen* OR preadolescen* OR youth* OR child* OR schoolchild* OR minors OR teen OR teens OR teenager* OR infan* OR toddler* OR pediatr* OR paediatr* OR puber* OR baby OR babies OR girl* OR boy* OR newborn* OR neonat* OR premature* OR pre-matur* OR kid OR kids OR underag* OR kindergar* OR pubescen* OR prepubesc* OR school* OR preschool* OR highschool* OR suckling OR PICU OR NICU OR PICUs OR NICUs):ab,ti,kw) NOT ([Conference Abstract]/lim OR [Conference Review]/lim)

***Medline***

((episcore* OR episign* OR ((methylation* OR epigenetic) ADJ6 (risk) ADJ6 (score* OR measure)) OR ((methylation*) ADJ6 (profile) ADJ6 (score*)) OR ((methylation* OR epigenetic*) ADJ3 (proxy)) OR ((DNAm OR DNA-methylation* OR polyepigenetic OR poly-epigenetic OR aggregate-methylation*) ADJ3 (score*)) OR aggregate-DNAm OR methscor* OR methylation-score* OR (MPS AND methylat*)).ab,ti,kf.) **AND** (exp Child/ OR exp Infant/ OR (juvenil* OR adolescen* OR preadolescen* OR youth* OR child* OR schoolchild* OR minors OR teen OR teens OR teenager* OR infan* OR toddler* OR pediatr* OR paediatr* OR puber* OR baby OR babies OR girl* OR boy* OR newborn* OR neonat* OR premature* OR pre-matur* OR kid OR kids OR underag* OR kindergar* OR pubescen* OR prepubesc* OR school* OR preschool* OR highschool* OR suckling OR PICU OR NICU OR PICUs OR NICUs).ab,ti,kf.) NOT (congres* OR abstract*).pt.

***Web of Science***

TS=(((episcore* OR episign* OR ((methylation* OR epigenetic) NEAR/5 (risk) NEAR/5 (score* OR measure)) OR ((methylation*) NEAR/6 (profile) NEAR/6 (score*)) OR ((methylation* OR epigenetic*) NEAR/2 (proxy)) OR ((DNAm OR DNA-methylation* OR polyepigenetic OR poly-epigenetic OR aggregate-methylation*) NEAR/2 (score*)) OR aggregate-DNAm OR methscor* OR methylation-score* OR (MPS AND methylat*))) **AND** ((juvenil* OR adolescen* OR preadolescen* OR youth* OR child* OR schoolchild* OR minors OR teen OR teens OR teenager* OR infan* OR toddler* OR pediatr* OR paediatr* OR puber* OR baby OR babies OR girl* OR boy* OR newborn* OR neonat* OR premature* OR pre-matur* OR kid OR kids OR underag* OR kindergar* OR pubescen* OR prepubesc* OR school* OR preschool* OR highschool* OR suckling OR PICU OR NICU OR PICUs OR NICUs))) NOT DT=(Meeting Abstract OR Meeting Summary)

***Google Scholar***

episcore|episign|episignature|'methylation|epigenetic risk score'|'methylation|epigenetic proxy'|'DNAm|polyepigenetic score'|'aggregate DNAm'|methscore juvenile|adolescent| child|children|infant|pediatric|paediatric|baby|babies|girl|boy|newborn|neonate

**Prisma checklist**

| **Section and Topic** | **Item #** | **Checklist item** | **Location where item is reported** |
| --- | --- | --- | --- |
| **TITLE** | | |  |
| Title | 1 | Identify the report as a systematic review. | Title |
| **ABSTRACT** | | |  |
| Abstract | 2 | See the PRISMA 2020 for Abstracts checklist. | Next page |
| **INTRODUCTION** | | |  |
| Rationale | 3 | Describe the rationale for the review in the context of existing knowledge. | Introduction |
| Objectives | 4 | Provide an explicit statement of the objective(s) or question(s) the review addresses. | Introduction |
| **METHODS** | | |  |
| Eligibility criteria | 5 | Specify the inclusion and exclusion criteria for the review and how studies were grouped for the syntheses. | 2. Systematic review of early life MPS research |
| Information sources | 6 | Specify all databases, registers, websites, organisations, reference lists and other sources searched or consulted to identify studies. Specify the date when each source was last searched or consulted. | 2. Systematic review of early life MPS research  **+** Acknowledgements **+** Supplementary Methods |
| Search strategy | 7 | Present the full search strategies for all databases, registers and websites, including any filters and limits used. | Supplementary Methods |
| Selection process | 8 | Specify the methods used to decide whether a study met the inclusion criteria of the review, including how many reviewers screened each record and each report retrieved, whether they worked independently, and if applicable, details of automation tools used in the process. | 2. Systematic review of early life MPS research |
| Data collection process | 9 | Specify the methods used to collect data from reports, including how many reviewers collected data from each report, whether they worked independently, any processes for obtaining or confirming data from study investigators, and if applicable, details of automation tools used in the process. | 2. Systematic review of early life MPS research  **+** Supplementary Methods |
| Data items | 10a | List and define all outcomes for which data were sought. Specify whether all results that were compatible with each outcome domain in each study were sought (e.g. for all measures, time points, analyses), and if not, the methods used to decide which results to collect. | **+** Table 1+ Supplementary Table 1 |
|  | 10b | List and define all other variables for which data were sought (e.g. participant and intervention characteristics, funding sources). Describe any assumptions made about any missing or unclear information. | **+** Table 1; Supplementary Table 1 |
| Study risk of bias assessment | 11 | Specify the methods used to assess risk of bias in the included studies, including details of the tool(s) used, how many reviewers assessed each study and whether they worked independently, and if applicable, details of automation tools used in the process. | Not part of this paper |
| Effect measures | 12 | Specify for each outcome the effect measure(s) (e.g. risk ratio, mean difference) used in the synthesis or presentation of results. | Not applicable |
| Synthesis methods | 13a | Describe the processes used to decide which studies were eligible for each synthesis (e.g. tabulating the study intervention characteristics and comparing against the planned groups for each synthesis (item #5)). | 2. Systematic review of early life MPS research |
|  | 13b | Describe any methods required to prepare the data for presentation or synthesis, such as handling of missing summary statistics, or data conversions. | Not applicable |
|  | 13c | Describe any methods used to tabulate or visually display results of individual studies and syntheses. | 3. Which MPSs are most commonly used in early life? **+** Figure 1 **+** Table 1 **+** Supplementary Table 1 |
|  | 13d | Describe any methods used to synthesize results and provide a rationale for the choice(s). If meta-analysis was performed, describe the model(s), method(s) to identify the presence and extent of statistical heterogeneity, and software package(s) used. | Not applicable |
|  | 13e | Describe any methods used to explore possible causes of heterogeneity among study results (e.g. subgroup analysis, meta-regression). | Not applicable |
|  | 13f | Describe any sensitivity analyses conducted to assess robustness of the synthesized results. | Not applicable |
| Reporting bias assessment | 14 | Describe any methods used to assess risk of bias due to missing results in a synthesis (arising from reporting biases). | Not applicable |
| Certainty assessment | 15 | Describe any methods used to assess certainty (or confidence) in the body of evidence for an outcome. | Not applicable |
| **RESULTS** | | |  |
| Study selection | 16a | Describe the results of the search and selection process, from the number of records identified in the search to the number of studies included in the review, ideally using a flow diagram. | 2. Systematic review of early life MPS research |
|  | 16b | Cite studies that might appear to meet the inclusion criteria, but which were excluded, and explain why they were excluded. | Not reported |
| Study characteristics | 17 | Cite each included study and present its characteristics. | Supplementary Table 1 |
| Risk of bias in studies | 18 | Present assessments of risk of bias for each included study. | Not reported (no formal risk-of-bias assessment described). |
| Results of individual studies | 19 | For all outcomes, present, for each study: (a) summary statistics for each group (where appropriate) and (b) an effect estimate and its precision (e.g. confidence/credible interval), ideally using structured tables or plots. | Not applicable |
| Results of syntheses | 20a | For each synthesis, briefly summarise the characteristics and risk of bias among contributing studies. | 3. Which MPSs are most commonly used in early life? **+** 4. What factors influence MPSs performance in early life? **+** 6. Discussion and outlook |
|  | 20b | Present results of all statistical syntheses conducted. If meta-analysis was done, present for each the summary estimate and its precision (e.g. confidence/credible interval) and measures of statistical heterogeneity. If comparing groups, describe the direction of the effect. | Not applicable |
|  | 20c | Present results of all investigations of possible causes of heterogeneity among study results. | Not applicable |
|  | 20d | Present results of all sensitivity analyses conducted to assess the robustness of the synthesized results. | Not applicable |
| Reporting biases | 21 | Present assessments of risk of bias due to missing results (arising from reporting biases) for each synthesis assessed. | Not applicable |
| Certainty of evidence | 22 | Present assessments of certainty (or confidence) in the body of evidence for each outcome assessed. | Not applicable |
| **DISCUSSION** | | |  |
| Discussion | 23a | Provide a general interpretation of the results in the context of other evidence. | 6. Discussion and outlook |
|  | 23b | Discuss any limitations of the evidence included in the review. | 6. Discussion and outlook |
|  | 23c | Discuss any limitations of the review processes used. | Discussion and outlook |
|  | 23d | Discuss implications of the results for practice, policy, and future research. | 6. Discussion and outlook |
| **OTHER INFORMATION** | | |  |
| Registration and protocol | 24a | Provide registration information for the review, including register name and registration number, or state that the review was not registered. | Registered with institution, not reported |
|  | 24b | Indicate where the review protocol can be accessed, or state that a protocol was not prepared. | Registered with institution, not reported |
|  | 24c | Describe and explain any amendments to information provided at registration or in the protocol. | None |
| Support | 25 | Describe sources of financial or non-financial support for the review, and the role of the funders or sponsors in the review. | Acknowledgements |
| Competing interests | 26 | Declare any competing interests of review authors. | Acknowledgements |
| Availability of data, code and other materials | 27 | Report which of the following are publicly available and where they can be found: template data collection forms; data extracted from included studies; data used for all analyses; analytic code; any other materials used in the review. | 5. DEMETRA: a Developmental Methylation Risk Atlas |

*From:*  Page MJ, McKenzie JE, Bossuyt PM, Boutron I, Hoffmann TC, Mulrow CD, et al. The PRISMA 2020 statement: an updated guideline for reporting systematic reviews. BMJ 2021;372:n71. doi: 10.1136/bmj.n71

| **Section and Topic** | **Item #** | **Checklist item** | **Reported (Yes/No)** |
| --- | --- | --- | --- |
| **TITLE** | | |  |
| Title | 1 | Identify the report as a systematic review. | Yes |
| **BACKGROUND** | | |  |
| Objectives | 2 | Provide an explicit statement of the main objective(s) or question(s) the review addresses. | Yes |
| **METHODS** | | |  |
| Eligibility criteria | 3 | Specify the inclusion and exclusion criteria for the review. | Yes |
| Information sources | 4 | Specify the information sources (e.g. databases, registers) used to identify studies and the date when each was last searched. | Yes |
| Risk of bias | 5 | Specify the methods used to assess risk of bias in the included studies. | No |
| Synthesis of results | 6 | Specify the methods used to present and synthesise results. | Yes |
| **RESULTS** | | |  |
| Included studies | 7 | Give the total number of included studies and participants and summarise relevant characteristics of studies. | Yes |
| Synthesis of results | 8 | Present results for main outcomes, preferably indicating the number of included studies and participants for each. If meta-analysis was done, report the summary estimate and confidence/credible interval. If comparing groups, indicate the direction of the effect (i.e. which group is favoured). | No |
| **DISCUSSION** | | |  |
| Limitations of evidence | 9 | Provide a brief summary of the limitations of the evidence included in the review (e.g. study risk of bias, inconsistency and imprecision). | Yes |
| Interpretation | 10 | Provide a general interpretation of the results and important implications. | Yes |
| **OTHER** | | |  |
| Funding | 11 | Specify the primary source of funding for the review. | No |
| Registration | 12 | Provide the register name and registration number. | No |

*From:*  Page MJ, McKenzie JE, Bossuyt PM, Boutron I, Hoffmann TC, Mulrow CD, et al. The PRISMA 2020 statement: an updated guideline for reporting systematic reviews. BMJ 2021;372:n71. doi: 10.1136/bmj.n71
