# Supplementary material for: Paediatric DNA methylation profile scores: a systematic review and open-source atlas
Source: eBioMedicine. 2026 May 22;128:106300. doi: 10.1016/j.ebiom.2026.106300 (PMC13224112; doi:10.1016/j.ebiom.2026.106300)

Overview of early life methylation profile score (MPS) research, its challenges, and the DEMETRA atlas

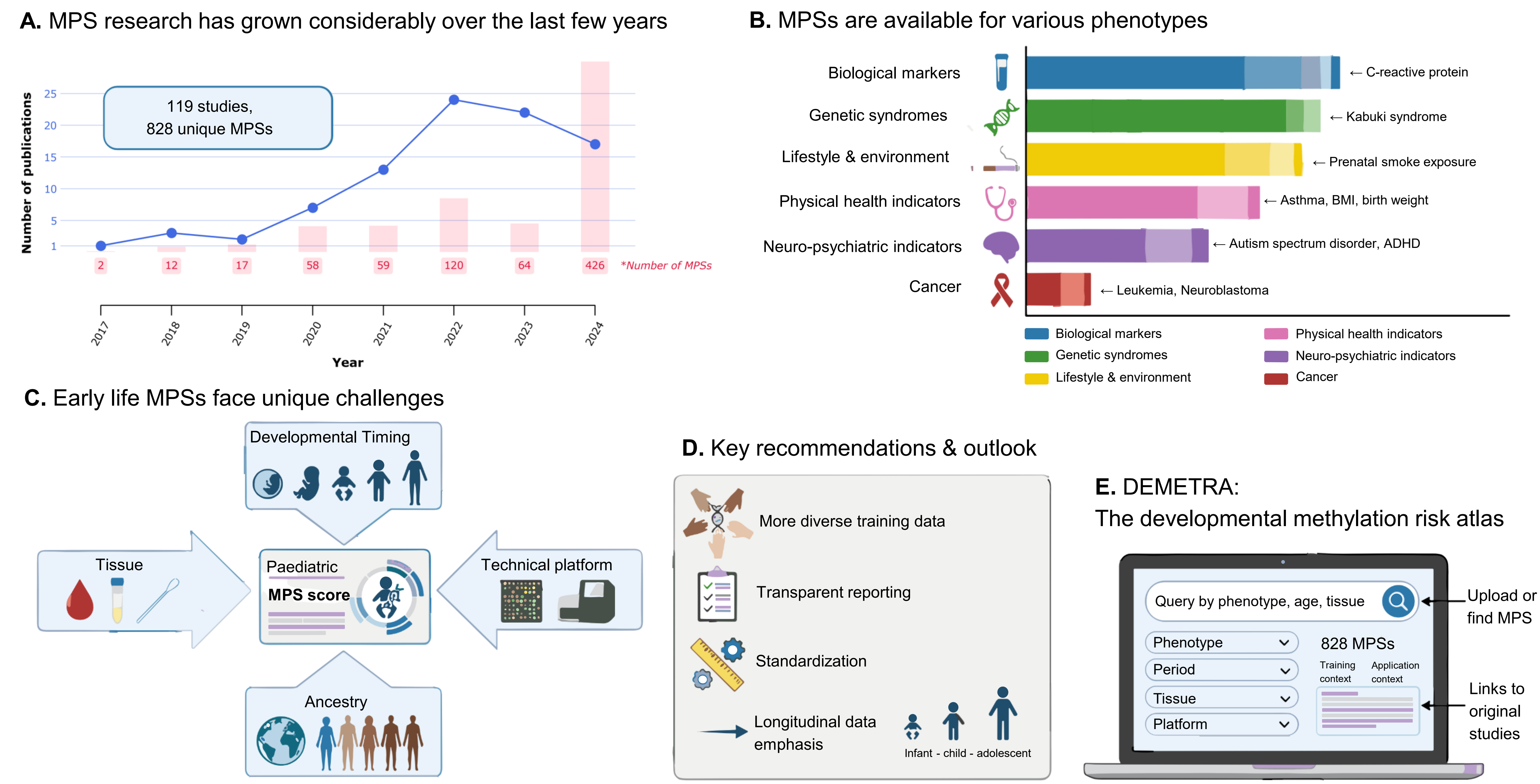

Supplement: Graphical abstract [file mmc3.pdf]
